# No differences in microbiome changes between anti‐adhesive and antibacterial ingredients in toothpastes during periodontal therapy

**DOI:** 10.1111/jre.12645

**Published:** 2019-03-09

**Authors:** Daniel Hagenfeld, Karola Prior, Inga Harks, Yvonne Jockel‐Schneider, Theodor W. May, Dag Harmsen, Ulrich Schlagenhauf, Benjamin Ehmke

**Affiliations:** ^1^ Department of Periodontology and Conservative Dentistry Muenster University Hospital Muenster Germany; ^2^ Department of Periodontology University Hospital Wuerzburg Wuerzburg Germany; ^3^ Society for Biometry and Psychometry Bielefeld Germany

**Keywords:** dental plaque, nonsurgical periodontal therapy, periodontal microbiota, subgingival plaque

## Abstract

**Aim:**

This subgroup analysis of a 12‐week randomized, double‐blind, and two‐center trial aimed to evaluate whether two different toothpaste formulations can differentially modulate the dental microbiome.

**Material and Methods:**

Forty one mild to moderate periodontitis patients used as an adjunct to periodontal treatment either a toothpaste with anti‐adhesive zinc‐substituted carbonated hydroxyapatite (HA) or with antimicrobial and anti‐adhesive amine fluoride/stannous fluoride (AmF/SnF_2_) during a 12‐week period. Plaque samples from buccal/lingual, interproximal, and subgingival sites were taken at baseline, 4 weeks after oral hygiene phase, and 8 weeks after periodontal therapy. Samples were analyzed with paired‐end Illumina Miseq 16S rDNA sequencing. The differences and changes on community level (alpha and beta diversity) and on the level of single agglomerated ribosomal sequence variants (aRSV) were calculated with analysis of covariance (ANCOVA) and likelihood ratio test (LRT).

**Results:**

Interproximal and subgingival sites harbored predominately *Fusobacterium* and *Prevotella* species associated with periodontitis, whereas buccal/lingual sites harbored mainly *Streptococcus* and *Veillonella* species associated with periodontal health. Alpha and beta diversity did not change noticeably differently between both toothpaste groups (*P* > 0.05, ANCOVA). Furthermore, none of the aRSVs showed a noticeably different change between the tested toothpastes during periodontal therapy (*P*_adj ._> 0.05, LRT).

**Conclusion:**

The use of a toothpaste containing anti‐adhesive HA did not induce statistically noticeably different changes on microbial composition compared to an antimicrobial and anti‐adhesive AmF/SnF_2_ formulation.

## 1 INTRODUCTION

The initiation and progression of caries and periodontitis, the most prevalent diseases of mankind, are closely associated with the establishment of disease‐promoting bacterial biofilms on tooth surfaces.^1^, ^2^ Their efficacious mechanical removal by properly performed oral hygiene therefore is generally regarded to be essential for predictable disease prevention.^3^ Furthermore, reduction in existing cleaning deficits by structured hygiene training instructions is an indispensable necessity. However, as approximately 30%‐60% of health information is forgotten within one hour^4^ and as not all affected patients may even have access to professionally guided oral hygiene training, antimicrobial agents are often added as toothpaste ingredients to level out insufficient mechanical cleaning efficacy. Their anti‐inflammatory efficacy in the treatment of gingivitis has been recently reviewed.^5^ The combination of amine fluoride and stannous fluoride (AmF/SnF_2_) showed antimicrobial and plaque‐reducing, that is, anti‐adhesive properties against in situ oral biofilms grown on intraoral splints.^6^ Likewise, studies with hydroxyapatite (HA) containing oral care products reported anti‐adhesive effects^7^, but observed no specific antimicrobial effects of the hydroxyapatite particles in situ.^8^ To evaluate those different formulations under clinical conditions, a randomized controlled study was conducted with stage I and II periodontitis patients^9^ using either a HA‐ or an AmF/SnF_2_‐containing toothpaste for 12 weeks while receiving periodontal therapy.^10^ Results of this study showed no differences between toothpastes in reducing the visible plaque on teeth or interfering with the de novo plaque formation. However, in this study, only quantitative plaque parameters were evaluated, so it remains open, if there are any compositional changes of bacteria within the dental plaque microbiome during the 12‐week study period.

Therefore, the objective of this study was to explore the composition of the whole microbiota in plaque samples taken by Harks et al^10^ using Illumina 16S rDNA sequencing to evaluate whether the previous observed similarity regarding quantitative plaque parameters between the two tested toothpastes (HA and AmF/SnF_2_) can also be found for the qualitative composition of the microbiota during periodontal therapy.

## 2 MATERIAL AND METHODS

### 2.1 Patient cohort

This is a metataxonomic sequencing analysis of a subsample of 41 patients from the already published clinical investigation of Harks et al^10^ registered at Clinical Trials.gov (NCT02697539). This clinical investigation was double‐blinded, randomized and had two participating centers: Dept. of Periodontology and Restorative Dentistry, University Hospital, Muenster, Germany, and Dept. of Periodontology, University Hospital, Wuerzburg, Germany. Inclusion criteria were pocket probing depths (PPD) of ≥4 mm at a minimum of four teeth (except third molars). Age ≥18‐75 years. Patients must have had at least 10 natural teeth (except third molars) and were nonsmokers. Exclusion criteria were known systemic diseases that may influence the periodontal conditions and also regular consumption of drugs that may interfere with periodontal conditions. Patients undergoing or requiring extensive dental or orthodontic treatment, were pregnant or breastfeeding were also excluded from the study. Furthermore, patients undergoing professional periodontal therapy during the 6 months prior to baseline and patients with periodontal pockets ≥6 mm in more than two sextants were excluded. The study was approved by the Ethics Committee of the Medical Faculty of the University of Wuerzburg, Germany (file # 2/11), and all participants gave their written informed consent. In this sub‐analysis, for each sequencing run, at least 10 patients from the study collective were selected consecutively. We conducted after each run an intermediate analysis according to the statistical analyses described below. Because between the 3rd and 4th sequencing run, no additional changes in outcomes were detected, we did not sequence the remaining patients from the full study because we did not expect any further changes.

### 2.2 Study design

**Figure 1:**
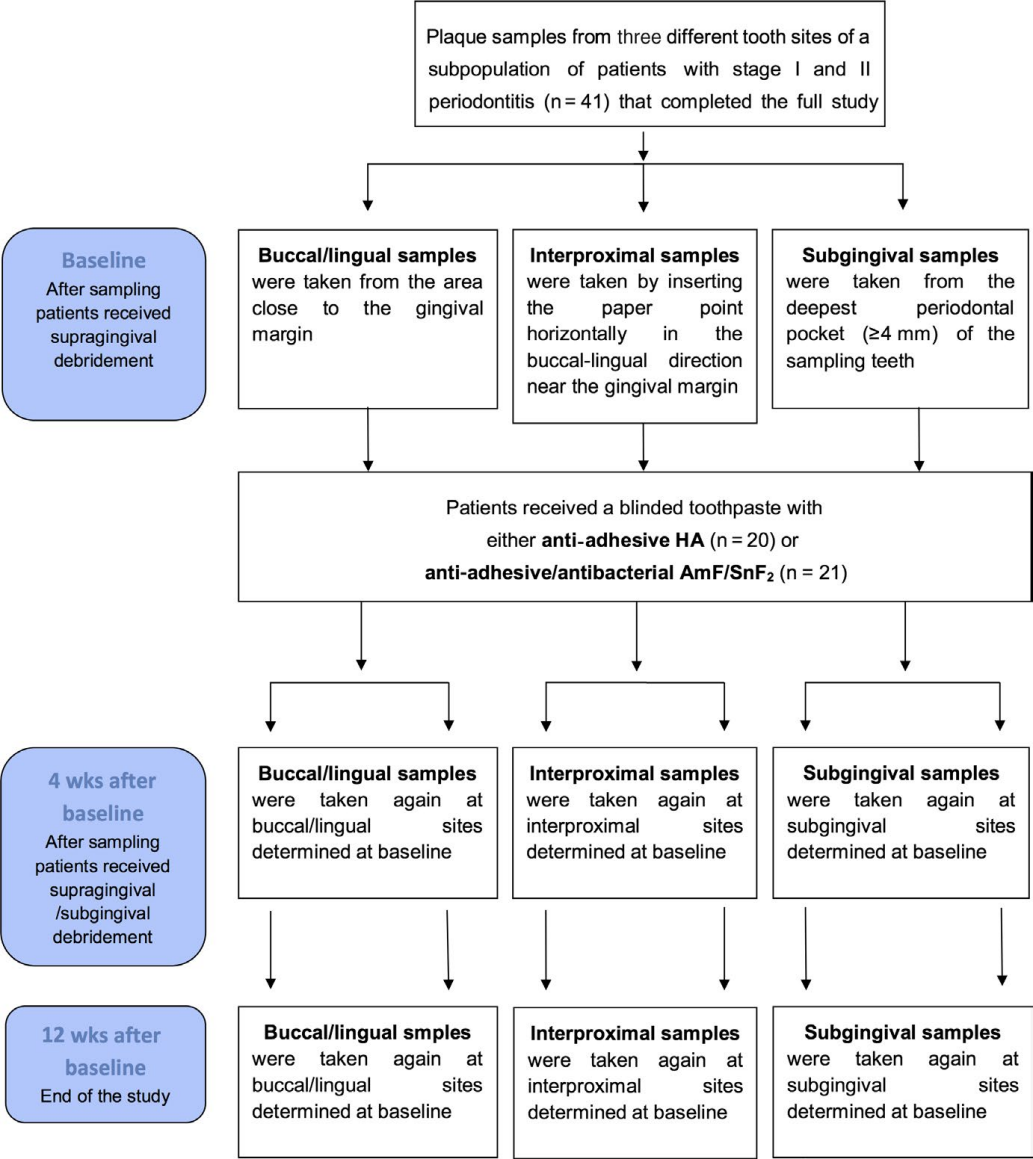
Flowchart of samples used in this study. Samples were taken from a larger finished study by Harks et al^10^ with 67 mild‐moderate periodontitis patients

At baseline, clinical periodontal examinations were done and the blinded toothpastes were dispensed. Patients received either a zinc‐substituted carbonated hydroxyapatite dentifrice (HA group, BioRepair, Wolff, Bielefeld, Germany) or a dentifrice containing an amine fluoride/stannous fluoride (AmF/SnF_2_ group, Meridol, CP GABA, Hamburg, Germany) with no further oral hygiene instructions. Thereafter, strict supragingival debridement was performed, as described previously.^10^ After 4 weeks, mechanical periodontal therapy was performed according to the at baseline recorded clinical measurements. All patients were advised to keep brushing their teeth exclusively with the originally provided toothpaste. Twelve weeks after baseline, that is, 8 weeks after periodontal therapy, clinical examinations were repeated and the study was ended (Figure 1).

### 2.3 Sampling procedures

Four sample teeth were selected randomly and equally distributed throughout the mouth, as described previously.^10^ Alternating, the most distal or the most mesial tooth in each quadrant with at least 1 site with PPDs of ≥4 mm was selected randomly for sampling. This was done to ensure a homogenous distribution of diseased sampling teeth in each patient. Samples were taken with sterile paper points (ISO45, Roeko Dental, Langenau, Germany) at each visit (baseline, 4 weeks, 12 weeks) at the same sampling sites before performing mechanical therapy. Buccal and lingual plaque samples (buccal/lingual) were taken from the area close to the gingival margin. Interproximal supragingival plaque samples (interproximal) were collected by inserting the paper point horizontally in the buccal‐lingual direction near the gingival margin. Subgingival plaque samples (subgingival) were taken from the deepest periodontal pocket (≥4 mm) of the sampling teeth. Paper points from the four corresponding sampling sites were pooled and stored at −20°C until further use.

### 2.4 DNA extraction, 16S rDNA amplification, and amplicon sequencing

Bacterial genomic DNA was isolated and purified with the QiaAmp Mini DNA‐Isolation Kit (Qiagen, Hilden, Germany), as described previously.^11^ Library preparation was performed with two rounds of amplification following the 16S metagenomics sequencing library preparation guide (Part # 15044223 Rev. B, Illumina GmbH, Munich, Germany).^11^ Up to 96 libraries were normalized and pooled for an Illumina MiSeq sequencing run using the Illumina MiSeq Reagent Kit version (v.) 3 with marginally overlapping 300 bp paired‐end reads.

### 2.5 16S rDNA sequence processing

Amplification primers were removed with Cutadapt v.1.8.1^12^ and reads that did not contain at least 10 bases of the adapter sequence or had an error rate above 0.2 in the adapter region were removed. Primer trimmed reads were submitted to the European Nucleotide Archive (http://www.ebi.ac.uk/ena/) of EMBL European Bioinformatics Institute under the study accession number PRJEB28345 (Table S1).

Trimmed (raw) reads were then processed using the R language environment v.3.5.0^13^ and RStudio v.1.1.447^14^, following the DADA2 v.1.8.0 workflow described by Callahan et al.^15^ Applied pipeline settings were explained in detail, before.^11^ Briefly, forward reads were truncated at position 260 and reverse reads at 190 onwards. Reads were denoised and those overlapping at least 15 bp were merged with no mismatch allowed. Ribosomal sequence variants (RSVs) were taxonomically assigned using a naive bayesian classifier and the Silva v.128 training set.^16^ Utilizing the R‐package phyloseq v.1.24.0^17^, the following sample specific details were combined: (a) all non‐chimeric RSVs along with their classification down to genus level and their abundance; (b) the phylogenetic neighbor‐joining tree^18^; and (c) the patient identifier, study center (Muenster or Wuerzburg), treatment group (HA or AmF/SnF_2_), treatment time points (baseline, 4 weeks or 12 weeks), and sampling site (buccal/lingual, interproximal, subgingival). To remove spurious RSVs, all variants occurring in two or less samples were removed and closely related RSVs were tree‐based agglomerated by a cophenetic distance smaller than *h* = 0.03 using single‐linkage clustering. Those agglomerated RSVs are designated as aRSVs hereinafter.

### 2.6 Statistical analysis of demographic, clinical, and microbial variables

All inferential statistics were intended to be exploratory instead of confirmatory. The *P*‐values were considered statistically noticeable if *P* ≤ 0.05. All continuous variables were reported as mean ± standard deviation.

Available demographic variables were age and gender. For clinical and inflammatory variables, the de novo plaque formation rate^10^ (PFR), gingival index^19^ (GI), plaque index^20^ (PI), bleeding on probing^21^ (BOP), pocket probing depth (PPD), recession depth (REC), and attachment level (AL) were utilized. Differences in the variables center and gender between HA and AmF/SnF_2_ group at baseline were tested using Fisher's exact test. Two‐sided Mann‐Whitney *U* tests were done for continuous clinical and demographic variables. Because of only exploratory analyses, no multiple testing corrections were applied here.

To allow for comparison of alpha diversity measurements between samples, reads were 100 times randomly subsampled to the level of the sample with the least number of reads (4204) with the command *phyloseq::rarefy_even_depth*. For measurement of richness, the number of observed aRSVs in each sample was determined^22^ by using the command *phyloseq::estimate_richness*. For beta diversity, a Bray‐Curtis distance matrix was created with the command *phyloseq::distance*. Analysis of variance (ANOVA) was performed for baseline values of alpha and beta diversity as dependent variables and treatment group and center as between‐subject factors and the sampling site (buccal/lingual, interproximal, and subgingival) as within‐subject factor. Analysis of covariance (ANCOVA) was performed to test for time effects at w4 and w12 on alpha and beta diversity using “intervention” as dependent variable, and “center,” as well as the baseline value (baseline) of the corresponding microbial variable as cofactors. To explore community structure and reduce dimensionality, a principal coordinates analysis (PCoA) was done with the Bray‐Curtis dissimilarity matrix by eliciting the commands: *phyloseq::ordinate* and *phyloseq::plot_ordination*.

The analysis of differential abundance of aRSVs was done with the R‐package DESeq2 v.1.20.0^23^ as previously described^11^ with following modifications: A likelihood ratio test (LRT) was performed to test for differentially changed aRSVs between groups at 4 and 12 weeks. The full model contained the factors treatment group, time point, site and center and the interaction term between treatment group and time point. The reduced model did not contain the interaction term. The false discovery rate was controlled by applying the Benjamini‐Hochberg procedure to adjust the *P*‐values.^24^ Effects on the counts of an aRSV were considered as noticeable if adjusted *P*‐value (*P*_adj_) ≤ 0.05. For descriptive analyses only, aRSVs were agglomerated on genus level with the command *phyloseq::tax_glom* and taxonomically labeled when possible. If such genera included species described by Sokransky et al^25^, this genus was allocated to the given complex, as previously described.^11^ All figures were created with the R‐package ggplot2.^26^

## 3 RESULTS

### 3.1 Demographical and clinical variables at baseline

The mean age of the study subpopulation was 54.86 ± 10.19 years with 25 females and 16 males included. Nineteen patients were from the Muenster center and 22 from the Wuerzburg center. No noticeable differences between HA and AmF/SnF_2_ group regarding age (HA: 54.22 years; AmF/SnF_2_: 55.46 years; *P* = 0.386, Mann‐Whitney *U* test), sex (HA: 9 females 11 males; AmF/SnF_2_: 16 females/5 males; *P* = 0.058, Fisher's exact test), and center distribution (HA: 10 Muenster/10 Wuerzburg; AmF/SnF_2_: 9 Muenster/12 Wuerzburg; *P* = 0.758, Fisher's exact test) were found. Moreover, no noticeable differences of the clinical and inflammatory variables were observed between both groups before therapy (Table 1).

**Table 1:** Clinical variables before therapy for the HA and AmF/SnF_2_ group

	HA (n = 20)	AmF/SnF_2_ (n = 21)	*P*‐value
PFR (%)	49.46 ± 16.88	52.01 ± 16.56	0.442
GI	1.12 ± 0.32	1.17 ± 0.53	0.886
PI (%)	63.77 ± 18.26	66.99 ± 19.31	0.602
BOP (%)	24.41 ± 25.39	20.11 ± 19.17	0.723
PPD (mm)	2.61 ± 0.48	2.57 ± 0.32	0.629
REC (mm)	0.37 ± 0.40	0.32 ± 0.31	0.784
AL (mm)	2.98 ± 0.70	2.89 ± 0.45	0.835

All variables represent whole mouth scores and are shown as mean ± standard deviation. *P*‐values were calculated using Mann‐Whitney *U* tests comparing the variables between both groups.

AL, attachment level; BOP, bleeding on probing; GI, gingival index; PFR. de novo plaque formation rate; PI, plaque index; PPD, pocket probing depth; REC, recession depth.

### 3.2 Bioinformatical preparation of plaque samples

Out of 369 plaque samples initially processed, 10 samples did not satisfy quality criteria. These samples were re‐introduced into an additional sequencing run, and the reads from repeated runs were merged. After repetition and merging, only two samples showed total read counts below 10 000 reads (9812 and 4204). However, those two samples were not excluded from further analysis because their saturation in rarefaction curves suggested sufficient sequencing depth.

### 3.3 Site‐specific composition of aRSVs at baseline and after 4 and 12 weeks

**Figure 2:**
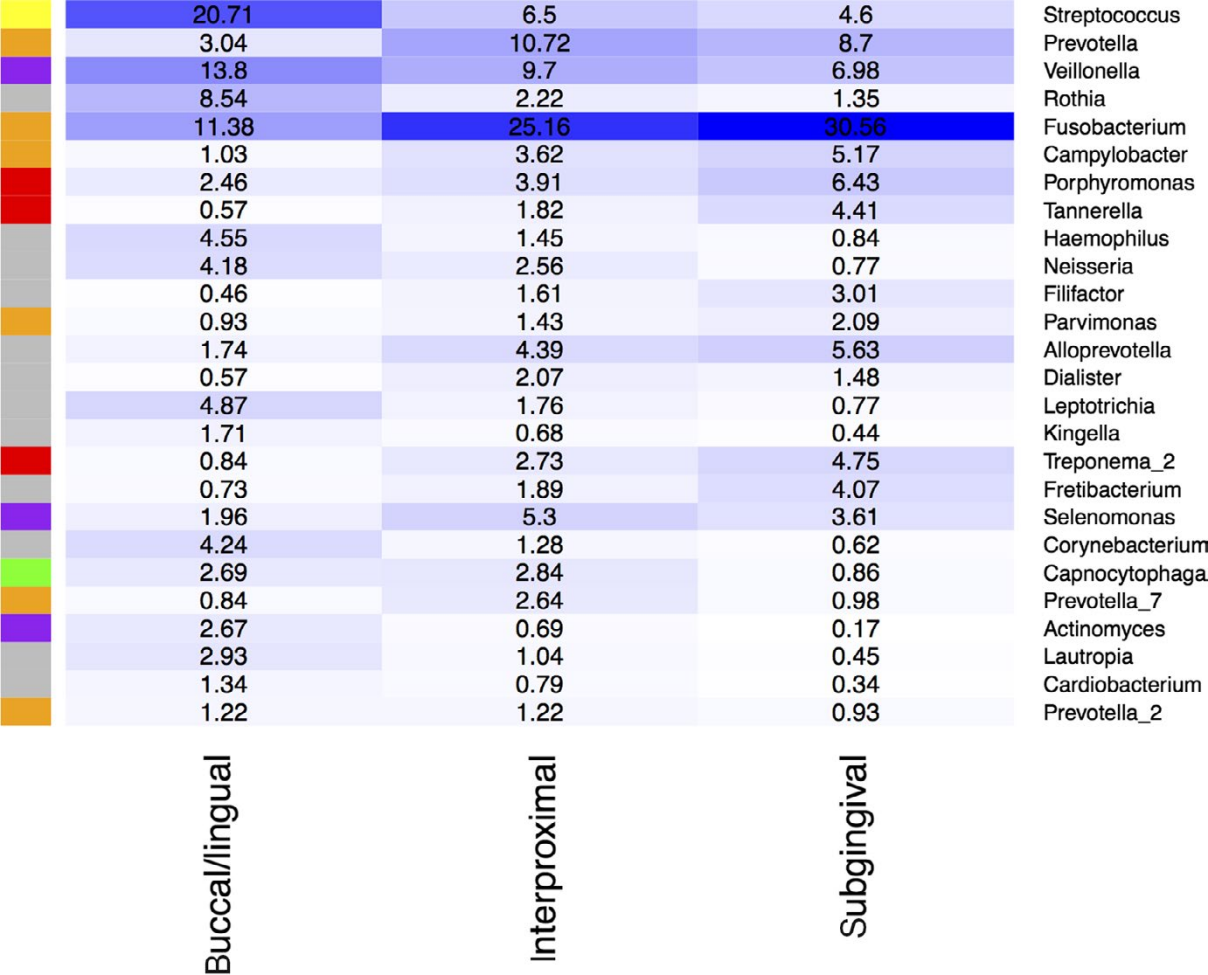
Heatmap of the distribution of highly abundant genera at baseline. Highly abundant genera were defined as having a minimal relative baseline abundance over all samples of at least 1% at one sampling site. On the right‐hand column are the taxonomic classifications down to genus level. On the left‐hand column are the color codes for the microbial complexes proposed by Socransky et al^25^; red and orange: species associated with periodontitis; purple, green, yellow: species associated with periodontal health; gray: no complex affiliation. The columns represent the three different dental sites: buccal/lingual, interproximal, and subgingival

After processing the raw reads with DADA2, 6387 non‐chimeric unique RSVs were found over all samples. By removing all RSVs occurring only in two or less samples, this number was reduced to 2993 RSVs. Tree‐based agglomeration of the remaining RSVs resulted in 393 aRSVs, which included 15 aRSVs taxonomically labeled as eukaryotes, archaea, or without any label on kingdom level. For further analysis, only the 378 bacterial aRSVs were selected that were categorized into 16 uniquely named taxa on phylum level and 117 on genus level. For inter‐sample comparisons of alpha diversity only, reads were normalized by randomly subsampling to the level of the sample with the least number of reads. This reduced the mean number of aRSVs per sample from 118.5 to 106.7. The most abundant genera before therapy at the buccal/lingual sites were *Streptococcus* with 20.71% mean relative read count before therapy (MRRC_b_) over both treatment groups*, Veillonella* (13.80% MRRC_b_), and *Fusobacterium* (11.38% MRRC_b_) (Figure 2). This was different to subgingival and interproximal sites where most abundant taxa were *Fusobacterium* (interproximal: 25.16% MRRC_b_; subgingival: 30.56 MRRC_b_), *Prevotella* (interproximal: 10.72% MRRC_b_; subgingival: 8.70 MRRC_b_), and *Veillonella* (interproximal: 9.70% MRRC_b_; sub: 6.98% MRRC_b_). No aRSV showed a noticeably different change (*P*_adj _> 0.05, LRT) between the HA and AmF/SnF_2_ group 4 and 12 weeks after therapy.

### 3.4 Alpha and beta diversity at baseline

**Figure 3:**
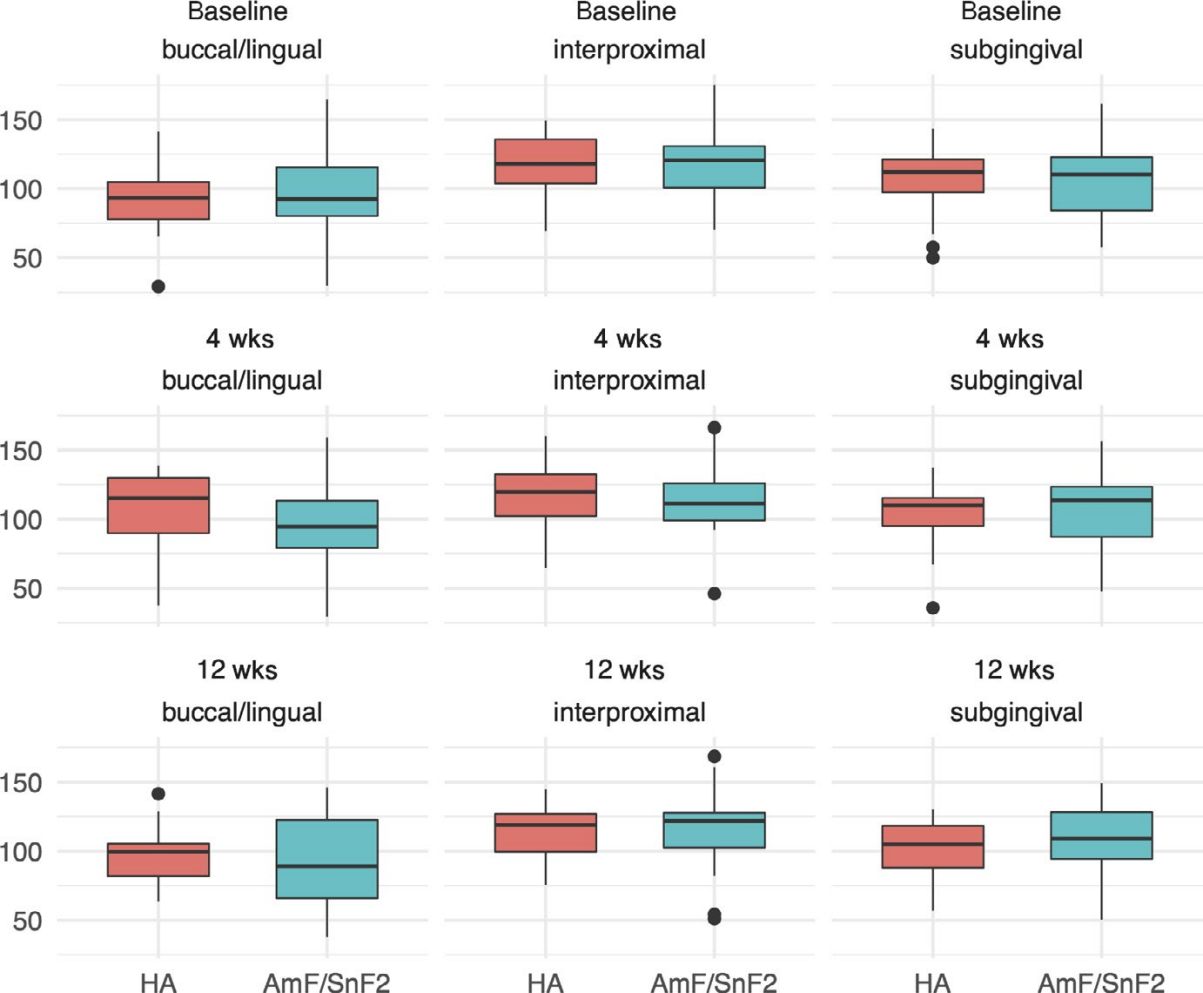
Boxplots of alpha diversity during periodontal therapy. Samples are visualized by boxes with whiskers and dots for outliers. Boxes are colored red for the HA and blue for the AmF/SnF_2_ group. Each row represents a different time point: before (baseline), 4 weeks after supragingival debridement, and 8 weeks after supragingival and subgingival debridement. Each column represents the site where the sample was taken from supragingival buccal and lingual (buccal/lingual), supragingival interdental (interproximal), subgingival interdental (subgingival) not accessible for daily oral hygiene. Mean species richness per treatment group at each time point and sample site was calculated after randomly subsampling aRSV counts with 100 cycles to the sample with the lowest reads

**Figure 4:**
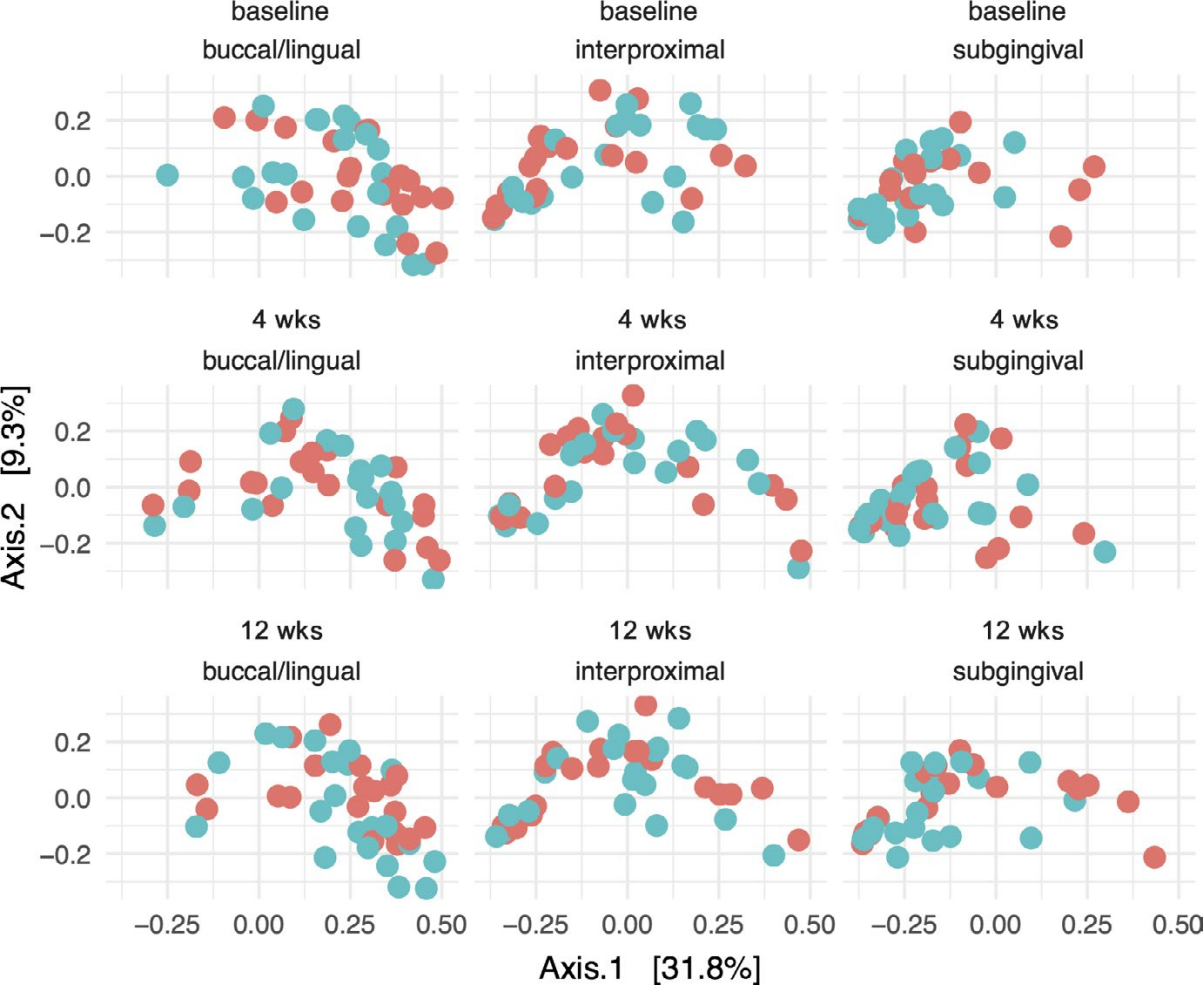
PCoA scatterplots of beta diversity during periodontal therapy. Samples are visualized by dots, which are colored red for the HA and blue for the AmF/SnF_2_ group. Each row represents a different time point: baseline, 4 weeks after supragingival debridement, and 8 weeks after supragingival and subgingival debridement. Each column represents the site where the sample was taken from supragingival buccal and lingual (buccal/lingual), supragingival interdental (interproximal), subgingival interdental (subgingival) not accessible for daily oral hygiene. The ordination was constructed using a Bray‐Curtis distance matrix. Principal component 1 (Axis 1) and principal component 2 (Axis 2) are plotted on the x‐ and y‐axes, respectively. The percentage of variation explained by the plotted principal coordinates is indicated on the axes

Alpha diversity showed noticeable center and site effects at baseline (ANOVA; center: *P* = 0.025 and site: *P* < 0.001). The normalized number (norm) of aRSVs was 98.49 ± 25.16 in the Muenster center compared to 114.50 ± 29.66 in the Wuerzburg center. At the buccal/lingual sites 96.10 ± 30.76, at the interproximal sites 118.65 ± 25.17, and at the subgingival sites 106.48 ± 25.90, norm aRSVs were found. There were no noticeable group differences at baseline between HA: 105.21 ± 26.28 norm aRSVs and AmF/SnF_2_: 108.86 ± 30.93 norm aRSVs (ANOVA group: *P* = 0.716) (Figure 3). Regarding beta diversity, there was a noticeable site effect (ANOVA site: *P* < 0.001), but no group or center effects at baseline (ANOVA group: *P* = 0.466, center: *P* = 0.577) (Figure 4).

### 3.5 Alpha and beta diversity changes before and after periodontal therapy

Alpha diversity did not change noticeably differently in the HA and AmF/SnF_2_ group during the 4 weeks before periodontal therapy (ANCOVA for buccal/lingual: HA: +13.41 ± 27.76 norm aRSVs vs AmF/SnF_2_: −1.23 ± 24.86 norm aRSVs, *P* = 0.082; interproximal: HA: −1.63 ± 19.98 norm aRSVs vs AmF/SnF_2_: −4.90 ± 17.90 norm aRSVs, *P* = 0.668; subgingival: HA: −2.47 ± 28.06 norm aRSVs vs AmF/SnF_2_: −0.46 ± 16.98 norm aRSVs, *P* = 0.710). There was also no noticeably different change 8 weeks after mechanical periodontal therapy (ANCOVA for buccal/lingual: HA: −10.27 ± 29.53 norm aRSVs vs AmF/SnF_2_: −4.44 ± 23.71 norm aRSVs, *P* = 0.270; interproximal: HA: −0.33 ± 19.03 norm aRSVs vs AmF/SnF_2_: +0.84 ± 15.19 norm aRSVs, *P* = 0.813; subgingival: HA: −3.32 ± 23.46 norm aRSVs vs AmF/SnF_2_: +2.55 ± 15.74 norm aRSVs, *P* = 0.242) (Figure 3).

Beta diversity did also not change noticeably differently in the test and control group 4 weeks before therapy (ANCOVA for buccal/lingual: *P* = 0.453; interproximal: *P* = 0.409; subgingival: *P* = 0.771) and 8 weeks after periodontal therapy (ANCOVA for buccal/lingual: *P* = 0.262; interproximal: *P* = 0.759; subgingival: *P* = 0.157) (Figure 4). All time‐dependent differences were analyzed for each site separately while controlling for the cofactor center to adjust for the reported baseline differences in alpha and beta diversity.

## 4 DISCUSSION

In this study, we used the Illumina MiSeq technology with paired‐end 300 bp sequencing reads to analyze the influence of anti‐adhesive HA compared to anti‐adhesive and antibacterial AmF/SnF_2_ on the buccal/lingual, interproximal, and subgingival microbiomes of 41 untreated stage I‐II periodontitis patients during periodontal therapy. We found no noticeable differential modulation of alpha or beta diversity and on single aRSV level between both tested toothpastes.

Nevertheless, we found microbial differences along habitational features at baseline. At all sample teeth, three different tooth sites were sampled: the supragingival buccal/lingual sites that are expected to be highly accessible for daily oral hygiene, the supragingival interproximal sites, expected to be poorly accessible for daily oral hygiene, and also, at subgingival sites expected to be not accessible for daily oral hygiene. Interestingly sub‐ and interproximal sites harbored similar proportions of *Fusobacterium* and *Prevotella* previously associated with periodontitis^25^ (Figure 2). These sites differed to the buccal/lingual sites that contained mainly *Streptococcus*,* Veillonella*, and *Rothia* previously associated with periodontal health.^25^ Those findings are partly supported by the study of Simon‐Soro et al^27^ who further distinguished lingual from buccal sites. They used 500 bp 454 pyrosequencing and found that especially *Streptococcus* was highly prevalent at exposed buccal and nearly absent in more covered lingual sites. Intra‐individual differences between lingual, buccal, and oral mucosa were also found using 400 bp 454 pyrosequencing and Illumina HiSeq 2500.^28^ Here regardless of tissue type (mucosal, palatal, dental), surface‐associated bacterial communities varied along an ecological gradient from covered to more exposed surfaces. Taken this together, the limited access of oral niches might influence the microbiome composition and improvement of homecare daily plaque removal.

### 4.1 No differential effects on microbiota composition between the tested toothpastes before periodontal therapy

Hydroxyapatite particles that adhered to oral bacteria have been firstly visualized in saliva samples of subjects using HA‐containing oral care products in an in situ study with intraoral splints.^7^ Furthermore, a solution of pure HA particles reduced the thickness of the plaque on intraoral splints equivalent to chlorhexidine digluconate. Interestingly, live/dead staining suggested that those HA‐coated bacteria stayed alive, so it could be assumed that HA particles themselves have only anti‐adhesive and no antimicrobial properties.^8^ However, our tested HA product also contained zinc, surfactants, and preserving agents that are potential antimicrobial agents.^10^, ^29^ Thus, the final mode of action of the tested HA toothpaste is still not conclusively clarified, and to our best knowledge, this is the first study evaluating the effect of a HA‐containing toothpaste on bacterial community composition at different dental sites before and after professional periodontal therapy.

In the first part of this study from baseline to 4 weeks, patients received the blinded toothpastes without further oral hygiene instructions in an “over‐the‐counter model” to observe a possible differential effect in microbiome modulation between the toothpastes alone. However, alpha and beta diversity did not change noticeably differently between both groups (Figures 3 and 4). Furthermore, also on aRSV level, no differential change between the two tested toothpastes was found. These findings are corroborated by quantitative microbial results from our previous clinical study.^10^ Here, no differential effects between HA and AmF/SnF_2_ on the ratio of aerobic/anaerobic culture colony forming units were observed between baseline and 4 weeks. Additionally, we found no intra‐group effects on tested microbiome parameters after 4 weeks in the HA as well as in the AmF/SnF_2_ group (Figures 3 and 4). This observation is also supported by another microbiota‐based study by Huang et al.^30^ Here, a decrease in alpha and beta diversity was found only, after applying additionally an AmF/SnF_2_‐containing mouthwash together with the AmF/SnF_2_ toothpaste.^30^

Although the fluoride ion of AmF/SnF_2_ itself has only limited antimicrobial effects, it has been proposed that the amine portion of AmF possesses surface‐active properties and can prevent bacterial adhesion as well as inhibit bacterial growth.^31^ Metal salts can be bactericidal against oral bacteria and also possess anti‐plaque activity and can inhibit bacterial enzymes.^29^ Van Loveren et al^32^ evaluated 39 bacterial species from supragingival plaque of 30 subjects (mean age 26.8 ± 7.3 years) with DNA‐DNA checkerboard. By observing different rinsing and brushing protocols, a general plaque‐reducing effect of AmF/SnF_2_ toothpaste and mouthwash compared to the fluoride‐free control was found. Additionally, in this study, the plaque composition changed six hours after the AmF/SnF_2_ usage to a less acidogenic phenotype.

Furthermore, other active agents in toothpaste formulations have been recently tested on oral microbiota composition. For example, studies evaluating toothpastes containing arginine reported a decrease in bacterial diversity and especially antimicrobial effects on *Streptococcus*.^33^, ^34^ Another study that evaluated a toothpaste containing lactoperoxidase, lysozyme, and lactoferrin^35^ reported a microbiome shift after 12 weeks usage in form of increasing number of bacterial species associated with periodontal health and decrease in periodontal disease associated species.

### 4.2 No differential effect between both toothpastes after mechanical periodontal therapy

In the second part of this study, patients were advised to keep using the given toothpastes for 8 weeks after mechanical periodontal therapy to observe potential differential effects during the recolonization of bacteria after therapy. While in Harks et al^10^ the visible plaque on teeth was reduced after periodontal therapy in both toothpaste groups, in our study, beta and alpha diversity remained largely unchanged after periodontal therapy (Figures 3 and 4). This is supported by similar findings of previous microbiota‐based studies.^11^, ^36^, ^37^, ^38^ However, those studies did not include different toothpaste formulations. Other studies found qualitative microbiota changes after periodontal therapy at very favorably responding sites.^39^, ^40^ According to our pooled and randomized sample strategy, we cannot separate the individual well responding from non‐responding sites, but moreover give insights into the global changes of the individual microbiota. Furthermore, these findings suggest that mechanical periodontal therapy might be able to reduce the total bacterial load, but not always induce a global compositional microbiome change in every patient. Alternatively, microbiome changes might represent primarily habitational changes, *that is*, decreasing pocket depth after periodontal therapy. Therefore, those changes would be more pronounced at initial deep sites, which were not investigated by our study. Irrespective of using solely anti‐adhesive or anti‐adhesive and antimicrobial agents in toothpastes, patients were clinically successfully treated.^10^

### 4.3 Limitations

When interpreting results of this study, the following limitations should be considered. No negative control toothpaste group without any measurable effect on bacteria was included. Therefore, we cannot assess the extent of the antimicrobial or anti‐adhesive effect for each toothpaste alone. The patient population was older than patients from the referenced toothpaste/rinsing studies above. And our patients were already performing adequate oral hygiene and had only limited signs of gingival inflammation. Furthermore, by including only patients with mild to moderate periodontitis, the observed improvements of pockets and plaque parameters are expected to be lower; therefore, habitational changes were moderate. This might have an influence on detecting changes in microbiome composition, after therapy. The known center effect from the main study^10^ was also observed in alpha diversity in this study and had to be considered statistically as a confounding cofactor. Additionally, DESeq2 does not allow to include random effects in the aRSV level analysis, for example, to account for dependencies between multiple samples from the same patient.

## 5 CONCLUSION

The use of a toothpaste containing anti‐adhesive HA did not induce noticeably different changes on microbial composition compared to anti‐adhesive and antimicrobial AmF/SnF_2_. Within the limitations of this study, our results suggest that the tested antibacterial and anti‐adhesive ingredients have similar impact on the dental microbiome during periodontal therapy.

## CONFLICT OF INTEREST

Microbiological samples used for this manuscript originated from an already finished and published clinical study, which was funded by Kurt Wolff GmbH, Bielefeld, Germany. Theodor W. May received fees for consulting (statistics) the Dr. Kurt Wolff GmbH (Bielefeld, Germany). No other external funding, apart from the support of the authors’ institution, was available for this study.

## References

[1] Manji F , Dahlen G , Fejerskov O . Caries and periodontitis: contesting the conventional wisdom on their aetiology. Caries Res. 2018;52(6):548‐564. 29694978 10.1159/000488948

[2] Mira A . Oral microbiome studies: potential diagnostic and therapeutic implications. Adv Dent Res. 2018;29(1):71‐77. 29355422 10.1177/0022034517737024

[3] Herrera D , Serrano J . Chemical supragingival plaque control (Chemical dental biofilm control) In: Lang NP, Lindhe J, eds. Clinical Periodontology and Implant Dentistry, 6th edn Chichester, UK: Wiley‐Blackwell; 2015:1480.

[4] Wilder RS , Bray KS . Improving periodontal outcomes: merging clinical and behavioral science. Periodontol 2000. 2016;71(1):65‐81. 27045431 10.1111/prd.12125

[5] Serrano J , Escribano M , Roldán S , Martín C , Herrera D . Efficacy of adjunctive anti‐plaque chemical agents in managing gingivitis: a systematic review and meta‐analysis. J Clin Periodontol. 2015;42:S106‐S138. 25495592 10.1111/jcpe.12331

[6] Auschill TM , Hein N , Hellwig E , Follo M , Sculean A , Arweiler NB . Effect of two antimicrobial agents on early in situ biofilm formation. J Clin Periodontol. 2005;32(2):147‐152. 15691343 10.1111/j.1600-051X.2005.00650.x

[7] Hannig C , Basche S , Burghardt T , Al‐Ahmad A , Hannig M . Influence of a mouthwash containing hydroxyapatite microclusters on bacterial adherence in situ. Clin Oral Investig. 2013;17(3):805‐814. 10.1007/s00784-012-0781-6 22782257

[8] Kensche A , Holder C , Basche S , Tahan N , Hannig C , Hannig M . Efficacy of a mouthrinse based on hydroxyapatite to reduce initial bacterial colonisation in situ. Arch Oral Biol. 2017;80:18‐26. 28364672 10.1016/j.archoralbio.2017.03.013

[9] Tonetti MS , Greenwell H , Kornman KS . Staging and grading of periodontitis: Framework and proposal of a new classification and case definition. J Clin Periodontol. 2018;45:S149‐S161. 29926495 10.1111/jcpe.12945

[10] Harks I , Jockel‐Schneider Y , Schlagenhauf U , et al. Impact of the daily use of a microcrystal hydroxyapatite dentifrice on de novo plaque formation and clinical/microbiological parameters of periodontal health. A randomized trial. PLoS ONE. 2016;11(7):e0160142. 27467683 10.1371/journal.pone.0160142 PMC4965058

[11] Hagenfeld D , Koch R , Jünemann S , et al. Do we treat our patients or rather periodontal microbes with adjunctive antibiotics in periodontal therapy? A 16S rDNA microbial community analysis PLoS ONE. 2018;13(4):e0195534. 29668720 10.1371/journal.pone.0195534 PMC5906003

[12] Martin M . Cutadapt removes adapter sequences from high‐throughput sequencing reads. EMBnet J. 2011;17(1):10.

[13] R Development Core Team . R: A language and environment for statistical computing; 2017 https://www.r-project.org. Accessed October 30, 2018.

[14] RStudio Team . RStudio: Integrated development environment for R; 2016 http://www.rstudio.com/. Accessed October 30, 2018.

[15] Callahan BJ , Sankaran K , Fukuyama JA , McMurdie PJ , Holmes SP . Bioconductor workflow for microbiome data analysis: from raw reads to community analyses. F1000Research. 2016;5:1492. 27508062 10.12688/f1000research.8986.1 PMC4955027

[16] Pruesse E , Quast C , Knittel K , et al. SILVA: a comprehensive online resource for quality checked and aligned ribosomal RNA sequence data compatible with ARB. Nucleic Acids Res. 2007;35(21):7188‐7196. 17947321 10.1093/nar/gkm864 PMC2175337

[17] McMurdie PJ , Holmes S . phyloseq: an R package for reproducible interactive analysis and graphics of microbiome census data. PLoS ONE. 2013;8(4):e61217. 23630581 10.1371/journal.pone.0061217 PMC3632530

[18] Saitou N , Nei M . The neighbor‐joining method: a new method for reconstructing phylogenetic trees. Mol Biol Evol. 1987;4(4):406‐425. 3447015 10.1093/oxfordjournals.molbev.a040454

[19] Löe H . The gingival index, the plaque index and the retention index systems. J Periodontol. 1967;38(6):610‐616. 10.1902/jop.1967.38.6.610 5237684

[20] O'Leary TJ , Drake RB , Naylor JE . The plaque control record. J Periodontol. 1972;43(1):38. 4500182 10.1902/jop.1972.43.1.38

[21] Lang NP , Joss A , Orsanic T , Gusberti FA , Siegrist BE . Bleeding on probing. A predictor for the progression of periodontal disease?. J Clin Periodontol. 1986;13(6):590‐596. 3489010 10.1111/j.1600-051x.1986.tb00852.x

[22] Kempton RA , Taylor LR . Models and statistics for species diversity. Nature. 1976;262(5571):818‐820. 958461 10.1038/262818a0

[23] Love MI , Huber W , Anders S . Moderated estimation of fold change and dispersion for RNA‐seq data with DESeq2. Genome Biol. 2014;15(12):550. 25516281 10.1186/s13059-014-0550-8 PMC4302049

[24] Benjamini Y , Hochberg Y . Controlling the false discovery rate: a practical and powerful approach to multiple testing. J R Stat Soc Ser B. 1995;57(1):289‐300.

[25] Socransky SS , Haffajee AD , Cugini MA , Smith C , Kent RL . Microbial complexes in subgingival plaque. J Clin Periodontol. 1998;25(2):134‐144. 9495612 10.1111/j.1600-051x.1998.tb02419.x

[26] Wickham H . Ggplot2: Elegant Graphics for Data Analysis, 1st edn New York: Springer‐Verlag; 2009.

[27] Simon‐Soro A , Tomas I , Cabrera‐Rubio R , Catalan MD , Nyvad B , Mira A . Microbial geography of the oral cavity. J Dent Res. 2013;92(7):616‐621. 23674263 10.1177/0022034513488119

[28] Proctor DM , Fukuyama JA , Loomer PM , et al. A spatial gradient of bacterial diversity in the human oral cavity shaped by salivary flow. Nat Commun. 2018;9(1):681. 29445174 10.1038/s41467-018-02900-1 PMC5813034

[29] Brading MG , Marsh PD . The oral environment: the challenge for antimicrobials in oral care products. Int Dent J. 2003;53(6 Suppl 1):353‐362. 14725379 10.1111/j.1875-595x.2003.tb00910.x

[30] Huang S , Li Z , He T , et al. Microbiota‐based signature of gingivitis treatments: a randomized study. Sci Rep. 2016;6:24705. 27094556 10.1038/srep24705 PMC4837389

[31] Baehni PC , Takeuchi Y . Anti‐plaque agents in the prevention of biofilm‐associated oral diseases. Oral Dis. 2003;9(Suppl 1):23‐29. 12974527 10.1034/j.1601-0825.9.s1.5.x

[32] van Loveren C , Gerardu VAM , Sissons CH , van Bekkum M , ten Cate JM . Effect of various rinsing protocols after use of amine fluoride/stannous fluoride toothpaste on the bacterial composition of dental plaque. Caries Res. 2009;43(6):462‐467. 20016176 10.1159/000264683

[33] Zheng X , He J , Wang L , et al. Ecological effect of arginine on oral microbiota. Sci Rep. 2017;7(1):7206. 28775282 10.1038/s41598-017-07042-w PMC5543048

[34] Koopman JE , Hoogenkamp MA , Buijs MJ , et al. Changes in the oral ecosystem induced by the use of 8% arginine toothpaste. Arch Oral Biol. 2017;73:79‐87. 27697693 10.1016/j.archoralbio.2016.09.008

[35] Adams SE , Arnold D , Murphy B , et al. A randomised clinical study to determine the effect of a toothpaste containing enzymes and proteins on plaque oral microbiome ecology. Sci Rep. 2017;7:43344. 28240240 10.1038/srep43344 PMC5327414

[36] Laksmana T , Kittichotirat W , Huang Y , et al. Metagenomic analysis of subgingival microbiota following non‐surgical periodontal therapy: a pilot study. Open Dent J. 2012;6:255‐261. 23341849 10.2174/1874210601206010255 PMC3547359

[37] Schwarzberg K , Le R , Bharti B , et al. The personal human oral microbiome obscures the effects of treatment on periodontal disease. PLoS ONE. 2014;9(1):e86708. 24489772 10.1371/journal.pone.0086708 PMC3906071

[38] Bizzarro S , Laine ML , Buijs MJ , et al. Microbial profiles at baseline and not the use of antibiotics determine the clinical outcome of the treatment of chronic periodontitis. Sci Rep. 2016;6:20205. 26830979 10.1038/srep20205 PMC4735321

[39] Soares GMS , Mendes JAV , Silva MP , et al. Metronidazole alone or with amoxicillin as adjuncts to non‐surgical treatment of chronic periodontitis: a secondary analysis of microbiological results from a randomized clinical trial. J Clin Periodontol. 2014;41(4):366‐376. 24834504 10.1111/jcpe.12217

[40] Shi B , Chang M , Martin J , et al. Dynamic changes in the subgingival microbiome and their potential for diagnosis and prognosis of periodontitis. MBio. 2015;6(1):e01926‐14. 25691586 10.1128/mBio.01926-14 PMC4337560

